# Media Discourse, Influence, and Reflection: Content Analysis and Text-Mining Study of Suicides and Homicides in Long-Term Care

**DOI:** 10.2196/59037

**Published:** 2025-04-28

**Authors:** Charlotte Wang, Hsiu-Ju Fang, Hsin-Yang Lu, Chen-Fen Chen

**Affiliations:** 1 Institute of Health Data Analytics and Statistics College of Public Health National Taiwan University Taipei Taiwan; 2 Master of Public Health Program College of Public Health National Taiwan University Taipei Taiwan; 3 Department of Long-Term Care National Taipei University of Nursing and Health Sciences Taipei Taiwan

**Keywords:** family caregiver, news framing, homicide, suicide, text mining

## Abstract

**Background:**

As populations age, the demand for long-term care services steadily increases. The effectiveness of government-promoted long-term care policies and the public’s access to relevant service information are demonstrably influenced by media representation. In addition, prior research has suggested that news framing can mitigate the negative influence (the Werther effect) with a more hopeful framing (the Papageno effect), thereby reducing the public’s susceptibility to negative news.

**Objective:**

This study investigates the phenomenon of suicides and homicides in long-term care reported in the news, in which family caregivers or care receivers died by suicide or homicide. We examined changes in the media’s reporting framework before and after the implementation of Taiwan’s Long-Term Care Plan 2.0 in 2017. We further examined the consistency between the content of news reports and the information provided by the media on long-term care services and suicide prevention (eg, hotlines).

**Methods:**

Content analysis and text-mining techniques were used to analyze 433 news reports covering 95 cases of suicides and homicides in long-term care in Taiwan from 2009 to 2021. A random-effects model was applied to examine term frequency transition post implementation.

**Results:**

The majority (>60%) of the cases involved family caregivers’ homicide-suicide. The term “family moral tragedy” has been replaced by “long-term care tragedy” in recent discourse. This shift is evident in a decline in the frequency of “family moral tragedy” since 2017, with usage decreasing by 32.4% in headlines and by 24% in news content. The term frequency of “care burden” has significantly increased from 0.0006 (SD 0.0008) to 0.017 (SD 0.0461; *t*_337_=3.006; *P*=.003). While linguistic characteristics of the content have remained consistent, there were statistically significant differences in medical and ethics-related terms. The media tends to provide more suicide prevention information (eg, hotlines; >50%), offering relatively limited coverage on long-term care services (<25%).

**Conclusions:**

The news media have the potential to change the public’s response to specific issues. Our findings suggest that government efforts to encourage media coverage of positive experiences with long-term care services can be a preventative measure against caregiving suicides and homicides. Moreover, government initiatives should focus on strengthening media publicity and enhancing media literacy within the long-term care sector. By empowering the media to provide readers with clear channels for seeking help, such as hotlines, the media will contribute positively to the mental health of family caregivers. Finally, an annual database on family caregiver homicide-suicide should be established. In that case, the government could identify potential risk factors and inform the formulation and revision of relevant policies and services via this database, ultimately contributing to preventing suicides and homicides in long-term care and achieving public health goals.

## Introduction

As the population ages, aging societies experience a corresponding rise in long-term care needs [1]. In response, governments typically implement long-term care policies and provide related services to address these emerging demands [2]. However, particularly in countries just beginning to confront aging-related challenges, public awareness and understanding of available policies and resources remain limited [3]. In such contexts, the news media can play a vital role not only by disseminating information about government policies and available services but also by shaping public perceptions, highlighting emerging issues, and facilitating informed public discourse [4,5]. Effective media coverage ensures that policies are widely recognized, better understood, and ultimately fully used by the population, thereby enhancing societal preparedness for demographic changes [6].

Family caregivers have long been regarded as essential “care resources” and gatekeepers for preventing suicide among care recipients [7]. However, they often endure significant stress, anxiety, depression, and sleep disorders due to their caregiving responsibilities, frequently leading to exhaustion [8-11]. Recently, increasing attention has been paid to the suicidal ideation and motivation of family caregivers [12-14]. Notably, there has been minimal research on their homicidal ideation. Such thoughts often stem from the absence of euthanasia options, compounded by the caregivers’ inability to cope with the suffering of care recipients and the relentless nature of caregiving [15,16]. Homicidal ideation among family caregivers represents a critical policy issue, as highlighted by caregiver homicide-suicides. Despite their severity, these events typically draw only fleeting public attention [17]. In government-related policy and social gerontology, research and discussion of these cases are lacking [7,18,19].

In 2011, a Taiwan newspaper reported a high-profile case of long-term care violence. An 84-year-old man, the sole caregiver for his wife who had Parkinson disease and multiple fractures from falls, drove a screwdriver into his wife’s forehead because he could not bear seeing her in pain from her illness [20]. The general public was drawn to this case and the media began reporting on family violence and suicide due to the stress of caring.

The Legislative Yuan passed the “Long-Term Care Services Act” in 2015, which defines long-term care as “the provision of life support, assistance, social participation, care, and related medical services to individuals with physical or mental disabilities lasting or expected to last for six months or more, based on the needs of the individuals or their family caregivers,” defining family caregivers as “main relatives or family members who provide regular care to disabled individuals within the family” and including among long-term care service delivery methods not only home-based, community-based, and institutional accommodation-based services but also “family caregiver support services.” The law also explicitly lists available “family caregiver support services,” indicating that it has begun considering the needs of family caregivers [21]. Taiwan’s long-term care policies and services have been developing for over a decade and were upgraded to the “Ten-Year Long-Term Care Plan 2.0” (Long-Term Care 2.0) in 2017, with a strong focus on developing home-based and community-based services [22]. Since 2017, government agencies have begun independently tracking the number of homicide cases attributed to caregiving burdens. The proportion of such cases has steadily increased, rising from 3.1% in 2017 to 17.1% in 2023 [23]. Moreover, possibly due to attention brought about by the law’s implementation, news articles on family caregiver suicides and homicides due to caregiving stress increased in 2018. This study aims to explore the phenomenon of homicides by family caregivers against care recipients and examine the characteristics of these cases.

While “long-term care” has become an important social topic and public policy priority, limited literature exists on how the media reports on issues related to family caregiver suicides and homicides. Framing theory, originating from discussions on how the news media constructs and represents reality, is a conceptual framework often used to analyze news reports. The media’s way of observing events—the frame—gives rise to a news report, which results from selecting and combining details that complete the story. Moreover, the framework of the news media may also change over time, and the information provided by the news media and the narrative framing can influence the public’s responses to specific issues [24,25]. In Taiwan, the promulgation of the “Long-Term Care Services Act” and promotion of Long-Term Care 2.0 have led to widespread use of the term “long-term care.” However, when the news equates “long-term care tragedy” with caregiving homicides, such as “Long-term Care Tragedy! Son Burns Himself and His Mother to Death After Allegedly Experiencing Despair as a Caregiver for Mother with Dialysis” [26] or “Tragic Long-term Care! Overwhelmed by Care Burden, Man Injects Excessive Medication, Commits Patricide” [27], readers focus on only the event itself. Since media coverage influences public response to long-term care issues, this study believes that it is necessary to analyze reports of suicides and homicides in long-term care, which include 3 types: the caregiver kills the care receiver and dies by suicide, the caregiver kills the care receiver without perpetrating suicide, and caregiver dies by suicide without involving the care recipient. Specifically, the study examines whether the media-reporting framework is consistent with the definition of long-term care in the “Long-Term Care Services Act” and whether the framework of media reports has changed with the promotion of new policies.

Furthermore, Jones [28] reminds us that suicide prevention should address the suicidal ideation of individuals with disabilities or impairments and the suicide contagion caused by how the media reports suicide, known as the Werther effect. Based on existing evidence, if suicide is repeatedly portrayed or interpreted as a normal response to stress, the likelihood of suicide imitation may increase. For instance, romanticization of spousal caregivers committing homicide-suicides as a way for couples to stay together even after death could exacerbate isolation and discourage individuals from seeking help. The World Health Organization has been urging global attention to suicide prevention since 1996 and has published a series of manuals for relevant personnel. These manuals emphasize that the news media should report on suicide with appropriate, accurate, and helpful information to prevent suicide. One such World Health Organization guideline is to “provide helpline numbers and community resources related to suicide prevention.” According to the Papageno effect, the media’s provision of opportunities for hope and relevant resources can positively impact suicide prevention [29]. Hence, this study explores whether the news media provides help-seeking information when reporting suicides and homicides, and whether the media’s reporting framework has undergone a significant change to promote Long-Term Care Plan 2.0.

In summary, this study investigates the phenomenon of suicides and homicides in long-term care reported in the news, in which family caregivers or care receivers died by suicide or homicide. We examined changes in the media’s reporting framework before and after the implementation of Taiwan’s Long-Term Care Plan 2.0 in 2017. We further examined the consistency between the content of news reports and the information provided by the media on long-term care services and suicide prevention (eg, hotlines).

## Methods

### Data

This study included all 95 cases of suicides and homicides in long-term care from 2009 to 2021 in Taiwan that we identified in the archive of the Taiwan Association of Family Caregivers, a nationwide social advocacy–oriented nonprofit organization dedicated to “family caregivers” [30] which, since 2009, has systematically collected “long-term care tragedy” news reports published on the web each day by conducting daily searches using keywords “long-term care,” “caregiving tragedies,” “elderly,” and “disabled people.”

Based on the 95 cases that were identified by carefully reading through the entire archive, we used the Google Search engine and news databases purchased by the National Taiwan University Library to manually review relevant news articles from various news media outlets in Taiwan, including news reports with text content, but excluding multimedia-only reports. Data were collected mostly from TV newscast transcripts, and newspaper articles, from the 8 mainstream media outlets. At the end of April 2022, 433 relevant news articles were collected, along with the name of the media source, reporter, report date, report content, social service information, and web page links for further analysis. Some cases were still under judicial review, and the news media continued to follow up and report on the judicial proceedings and outcomes. Relevant news articles related to these cases were included in this research. These news reports are public information, but all personally identifiable information has been redacted or anonymized.

### Ethical Considerations

This study was conducted in accordance with the ethical guidelines and regulations approved by the research ethics committee of National Taiwan University (NTU-REC nos. 201905ES111 and 202205ES143). The authors attest that the work was conducted by the ethical standards of the responsible committee on human experimentation (institutional and national) and with the Declaration of Helsinki in 1975.

### Content Analysis and Data Preprocessing

Content analysis [31] and text-mining techniques [32] were used to analyze the 433 news reports, including trend analysis of the number of cases and the number of news items, case information reported in the news, the media’s reporting framework, and social service information provided by the media.

Preprocessing of text data involved the following steps. The CKIP Tagger [33,34], an open-source Chinese word segmentation, part-of-speech tagging, and named-entity recognition system, was used to tokenize the 433 news reports, resulting in 11,677 terms. The frequency of each term appearing in each report was calculated, and a term document matrix was derived, with each cell representing the frequency of a single term in a specific report. Terms without apparent meaning, such as stop words, linking words, and particles, were removed; collocations were integrated into single words; and synonymous terms were replaced with single terms. This resulted in 6322 terms. The same data cleaning and preprocessing procedure was applied to news headlines, resulting in 911 terms.

### Statistical Analysis

We applied text-mining techniques to explore hidden information within the text and examine changing trends in the media’s reporting framework and incidence of suicides and homicides in long-term care. Because Taiwan’s long-term care system achieved a breakthrough in 2017 with the implementation of Taiwan’s Long-Term Care 2.0, we used this year as a dividing point to calculate the frequency of each term in each news item. We examined changes in term use frequency before December 31, 2016, and after January 1, 2017, to gain an understanding of the changes in vocabulary use trends. In addition, a random-effects model was applied to examine whether there were statistically significant differences between the average term frequencies used before 2017 and after. Moreover, we selected keywords related to long-term care to investigate the case types, associations, changes, and trends in long-term care tragic events, as well as the knowledge process and implicit information related to the social system.

## Results

### Basic Information of Suicides and Homicides in Long-Term Care in the News

We analyzed the trend of 433 news articles related to suicides and homicides in long-term care, covering 95 cases from 2009 to 2021. Of the 95 cases, 39 involved the deaths of both the family caregiver and the care recipient, and 56 cases involved the death of either the family caregiver or the care recipient (Table 1). According to news reports from 2009 to 2021, the highest number of cases was in 2018, with 20 cases, while the highest number of news reports was in 2019, with 71 reports.

We categorized the news into three types: (1) current news (news published immediately after the cases occurred), (2) investigation and judgment (news following judicial investigations or the announcement of trial outcomes), and (3) retrospective review (news reviewing previously occurring cases) (Table 2). Based on the types of cases and the time of the news reports, it was found that 7 cases (7.4%) garnered media attention only during the judgment stage. This indicates that some cases were not reported by the media when they happened but rather only when they were in the prosecutorial investigation or judicial trial stages. Analysis of the news content and time of publication found that when a family caregiver died by homicide-suicide, that is, when both parties died and the caregiver was not involved in the role of “defendant,” the “legal prosecution” aspect disappeared. Because such cases do not enter the judicial process, they result in fewer subsequent news reports. In other words, if government agencies or organizations that focus on this issue analyze trends in long-term care based solely on the news, they may overlook cases that have not been reported, leading to an underestimation of the incidence of suicides and homicides in long-term care. They may also overlook the potential social issues related to homicide-suicide cases among family caregivers.

However, no official government department statistics are available to accurately determine the incidence of such cases, making it difficult to ascertain the actual incidence rate and, thus, discuss the relationship between the actual incidence rate and the number of news articles.

**Table 1:** Annual number of suicides and homicides in long-term care reported in 2009-2022: types of cases (N=95).

Year	Cases in which both the caregiver and the care recipient died, n	Cases in which either the caregiver or the care recipient died, n	Total, n
2009	0	1	1
2010	0	1	1
2011	1	0	1
2012	6	0	6
2013	3	4	7
2014	7	2	9
2015	1	6	7
2016	7	7	14
2017	3	7	10
2018	6	14	20
2019	2	5	7
2020	1	5	6
2021	2	4	6
2022	0	0	0
Total	39	56	95

**Table 2:** Annual number of suicides and homicides in long-term care reported in 2009-2022: types of news article (N=433).

Year	Current news, n	Investigation and judgment, n	Retrospective review, n	Total, n
2009	3	0	0	3
2010	0	1	0	1
2011	3	3	1	7
2012	17	0	0	17
2013	10	0	0	10
2014	27	3	2	32
2015	19	9	0	28
2016	55	5	0	60
2017	23	4	0	27
2018	46	9	0	55
2019	49	21	1	71
2020	36	21	2	59
2021	39	14	3	56
2022	0	4	3	7
Total	327	94	12	433

### Analysis of Case Information in the News

Table 3 shows background characteristics of the 95 cases. Most family caregivers were men (63/99, 63.6%), and most care receivers were women (62/99, 62.6%). In terms of the type of relationship between caregivers and recipients, adult-child caregivers were the majority (35/102, 34.3%), followed by spouse caregivers (32/102, 31.4%), parent caregivers (19/102, 18.6%), and sibling caregivers (9/102, 8.8%). The most common case type involved family caregivers perpetrating homicide and subsequently perpetrating suicide (47/95, 49.4%), followed by family caregivers perpetrating homicide without subsequent suicide (25/95, 26.3%) and family caregivers perpetrating suicide independently (11/95, 11.6%). In summary, the combined percentage of cases involving family caregivers perpetrating homicide and subsequently perpetrating suicide, or perpetrating suicide on their own, exceeds 61%. This underscores the need for suicide prevention authorities to address this emerging issue proactively.

Moreover, 57.9% of news reports explicitly mentioned care recipient disabilities or dementia. The most common terms used were “mobility impaired” and “wheelchair-bound,” with fewer instances describing activities of daily living such as “feeding assistance required,” “bathing assistance needed,” or “urinary and fecal incontinence.” In other news, although it was mentioned that family caregivers felt overwhelmed or burdened by long-term care responsibilities, descriptions of care recipients’ physical and mental condition focused on disease status, stroke being the most frequently mentioned, followed by intellectual disability.

Furthermore, many news reports did not mention any use of social services by care recipients (39.4%). Whether this was a reporting failure or that care recipients did not apply for or use long-term care services is difficult to determine. The most used social service reported was financial assistance (13.5%), while a significant portion used no services (12.5%). Other reported service types involved live-in migrant care worker services (10.6%), residential facility services (8.7%), and home-based services (2.9%). A relatively small amount of news coverage including mention of Long-Term Care 2.0 services was reported.

**Table 3:** Background characteristics of the cases (N=95).

Characteristics		Cases, n (%)
**Sex of family caregivers^a^**		
	Male	63 (63.6)
	Female	36 (36.4)
**Sex of recipient^a^**		
	Male	37 (37.4)
	Female	62 (62.6)
**Relationship between family caregiver and recipient^b^**		
	Adult child caregiver	35 (34.3)
	Spouse caregiver	32 (31.4)
	Parent caregiver	19 (18.6)
	Sibling caregiver	9 (8.8)
	Other caregiver relationship	7 (6.9)
**Survival outcomes of both family caregivers and care recipient**		
	Caregiver dies by suicide after killing care recipient	47 (49.4)
	Caregiver kills care recipient without perpetrating suicide	25 (26.3)
	Caregiver dies by suicide without involving care recipient	11 (11.6)
	Others	12 (12.6)
Care recipients with disabilities or dementia mentioned in the news, n (%)		55 (57.9)
**Service use of care recipients^c^**		
	Unspecified service usage	41 (39.4)
	Financial assistance	14 (13.5)
	No service use	13 (12.5)
	Foreign caregiver employment	11 (10.6)
	Residential facility services	9 (8.7)
	Home-based services	3 (2.9)
	Others	13 (12.5)

^a^Among 95 cases, 4 cases had 2 caregivers, 2 cases had 2 or more care recipients, and 1 case was mutual caregiving between individuals.

^b^Among 95 cases, 6 cases had 2 relationships between family caregivers and recipients.

^c^Among 95 cases, 5 cases used 2 services and 2 cases used 3 services.

### Investigation of the Media’s Reporting Framework

**Figure 1:**
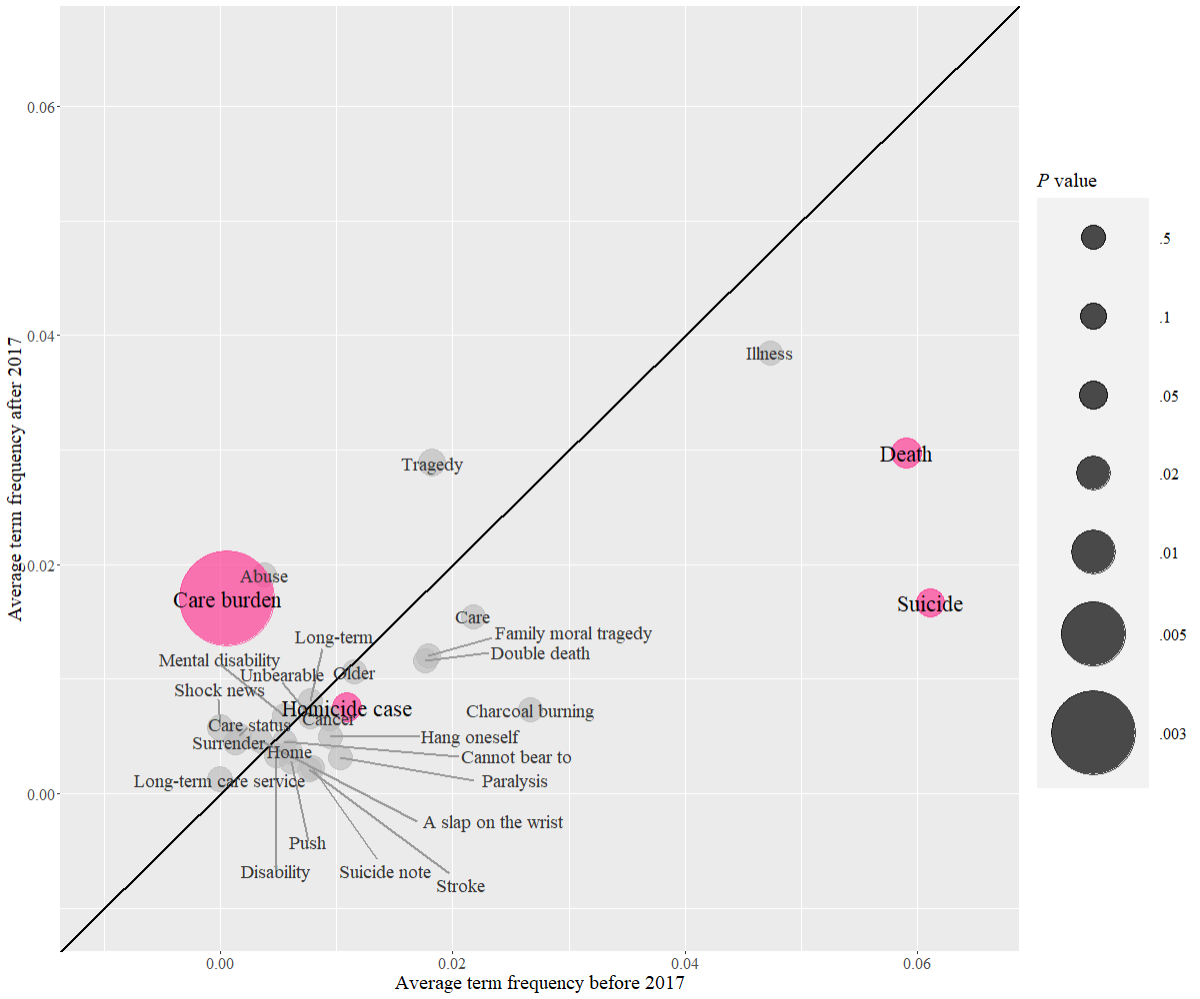
Analysis of the difference in the average term frequency of selected vocabulary used in news headlines before December 31, 2016, and after January 1, 2017, with the selection condition that the term appears in more than 10 news articles. The 45-degree line is a reference, representing equal use frequency before and after 2017. If a term appears in the lower-right quadrant, it indicates a higher average frequency before 2017. Conversely, if it appears in the upper-left quadrant, it suggests a higher average frequency after 2017. The further a point is from the 45-degree line, the more significant the difference in term frequency before and after 2017. The size of each circle represents the trend of significance testing (*P* value) of term frequency before and after 2017 based on the random-effects model. Larger circles indicate smaller *P* values, with magenta circles representing statistically significant differences (*P*<.05).

Using the implementation of Taiwan’s Long-Term Care 2.0 in 2017 as a dividing point in time, we investigated changes in the media’s reporting framework and shifts in vocabulary use, based on the headlines and contents of the reports (Multimedia Appendix 1). Figure 1 illustrates the analysis of differences in average term frequency (aTF) for selected vocabulary in news headlines before and after January 1, 2017. We observed that the term “family moral tragedy” is far from the 45 reference line and positioned in the lower-right quadrant, indicating a higher average frequency before 2017 (aTF=0.0179 before 2017 vs aTF=0.0121 after 2017). In contrast, the term “care burden” shows a significantly higher average frequency after 2017 (aTF=0.0006 before 2017 vs aTF=0.017 after 2017). This indicates that the framing in news headlines has shifted from stories of “family moral tragedies” to “long-term care tragedies.” In addition, use of the term “illness” is consistently higher before and after 2017 (aTF=0.0474 and aTF=0.0385, respectively) than use of terms such as “care status” (aTF=0.0016 before 2017 and aTF=0.005 after 2017) and “long-term care service” (aTF=0 before 2017 and aTF=0.0012 after 2017), indicating that the news often emphasizes the medical situation of the care receiver rather than the need for “long-term care.”

**Figure 2:**
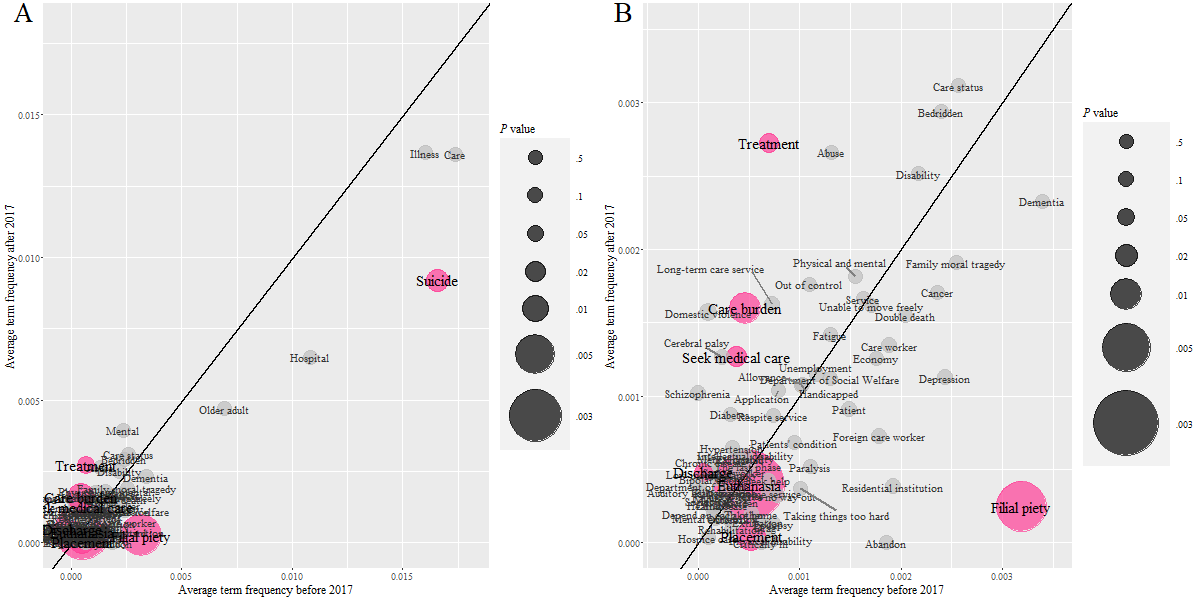
Analysis of the difference in the average term frequency of selected vocabulary used in news articles before December 31, 2016, and after January 1, 2017. (A) Normal size. (B) Zoomed-in version. The solid black line is a reference line at a 45-degree angle, representing equal use frequency before and after 2017. If a term appears in the lower-right quadrant, it indicates a higher average frequency before 2017. Conversely, if it appears in the upper-left quadrant, it suggests a higher average frequency after 2017. The further a point is from the 45-degree line, the more significant the difference in term frequency before and after 2017. The size of each circle is inversely proportional to the *P* value obtained from the hypothesis testing on term frequency before and after 2017 based on the random-effects model. Larger circles indicate smaller *P* values, with magenta circles representing statistically significant differences (*P*<.05).

We selected the 84 terms most relevant to “long-term care” and “disease” for further investigation, examined differences in term frequency before and after 2017, and analyzed changing trends in language usage within the news content. Figure 2 shows differences in aTF of vocabulary used in news reports before and after 2017. As shown in Figure 2A, the term “care” had the highest term frequency, approximately 0.013. Most terms occurred far less frequently than “care,” resulting in hard-to-identify terms in the lower-left quadrant; the magnified lower-left quadrant is shown in Figure 2B to address this. From Figures 2A and B, it is evident that the terms with statistically significantly different frequencies, such as “filial piety” (*t*_337_=–2.849; *P*=.005), “seek medical care” (*t*_337_=2.239; *P*=.03), and “treatment” (*t*_337_=2.133; *P*=.03), are most related to medical care and ethical considerations within social norms. Use of terms associated with long-term care services, such as “respite service” (*t*_337_=0.029; *P*=.98), “home service” (*t*_337_=–1.921; *P*=.06), “long-term care service” (*t*_337_=1.381; *P*=.17), and “care status” (*t*_337_=1.040; *P*=.30), was not significantly different before and after 2017 (Multimedia Appendix 1). This analysis provides insights into the nuanced changes and patterns in vocabulary use, particularly terms describing long-term care and disease. The findings suggest that specific terms reflect enduring cultural and ethical values. In contrast, terms directly related to long-term care services exhibited no significant shifts in use frequency between the specified periods.

Examination of the term “long-term care” in news headlines and content revealed exciting patterns. Within the news articles, the term “long-term care” was typically used to describe the care status of the individuals involved. Notably, after 2017, a significant increase in the emphasis on “care burden” occurred within news reports and the aTF of “care burden” (mean 0.017, SD 0.0461) in the headlines was considerably higher than that of “care status” (mean 0.005, SD 0.0265) and “long-term care service” (mean 0.0012, SD 0.0118). According to this, the implementation of Long-Term Care 2.0 had not yet established a strong connection between reported cases and long-term care services in the news media. Furthermore, media headlines often emphasized stress as a promotional angle, conveying tragic events resulting from caregiving stress without providing the audience, including family caregivers, alternative methods, or strategies for alleviating stress. Also, journalists primarily used “long-term care” as an adjective or verb to describe the care status of a case, rarely specifying its duration, instead of treating “long-term care” as a noun and connecting it to services provided by the government or caregiving needs of the case.

### Exploration of Disparities in the Provision of Social Service Information in the News

**Figure 3:**
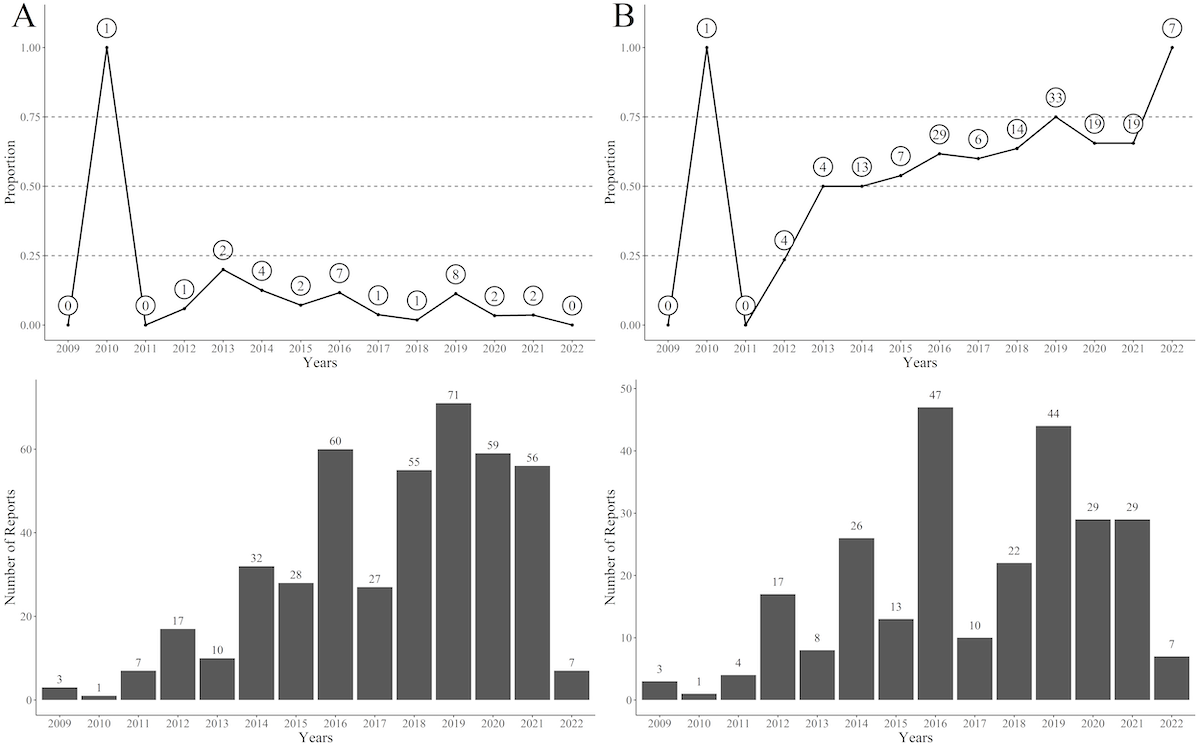
The trend of the number of news articles and social service information provided in the news about “long-term care tragedies” (2009-2022). (A) Long-term care services information. (B) Suicide prevention information. The bar chart represents (A) the total number of news articles about “long-term care tragedies” each year and (B) the number of news articles on “long-term care tragedies” that involve suicide. On the line graph, the numbers within the circles indicate the number of news articles each year that included long-term care information or suicide prevention information, and the y-axis represents the proportion of long-term care information or suicide prevention information provided, calculated by dividing the number within the circle by the total number of news articles for that year.

Despite their headlines, news reports usually do not provide social service information relevant to preventing suicides and homicides in long-term care, most pertinently long-term care services and suicide prevention information. Therefore, we analyzed the news’ inclusion of this information. We selected the news reports containing suicide-related vocabulary, such as “suicide,” “hanging,” “jumping,” and “carbon monoxide poisoning,” and calculated the number of news articles each year mentioning suicidal ideation or behavior. Figure 3 shows a trend analysis of whether the news stories provided corresponding social service information. For instance, Figure 3A shows that year 2016 shows 60 news articles about suicides and homicides in long-term care, with 11.7% (7/60) of them providing long-term care information. In contrast, Figure 3B shows 47 news articles about suicides and homicides in long-term care in 2016 that mentioned cases of suicidal thoughts or behaviors, among which 61.7% (29/47) offered suicide prevention information.

Comparing Figures 3A and B, from 2012 to 2022, the proportion of reports providing suicide prevention information remained roughly 50%, while the proportion delivering long-term care information was consistently below 25%. Some reports included social service information unrelated to their content, such as antibullying hotlines. Examination of the news’s social service information indicates that their grasp on suicide-related issues is well-defined and that, compared with media advocacy for long-term care services, government agencies may have a stronger presence in promoting suicide prevention. However, this also raises the possibility that the news media may lack a comprehensive understanding of long-term care information, resulting in an information presentation prioritizing suicide prevention over long-term care.

Since the news media serves as the primary source of information for most people, failure to provide long-term care service information while including social service information irrelevant to the particulars of the story might result in people in need of long-term care and experiencing similar difficulties accessing helpful information, increasing the burden of family caregiving.

## Discussion

### Principal Findings

The study analyzed 433 news reports covering 95 cases of suicides and homicides in long-term care in Taiwan between 2009 and 2021. A significant majority (>60%) of these cases involved family caregiver homicide-suicides. The most prevalent caregiver-recipient relationships were adult-child caregivers (35/102, 34.3%) and spouse caregivers (32/102, 31.4%). A notable shift in terminology occurred following the 2017 implementation of the Long-Term Care Plan 2.0, with news headlines and content transitioning from “family moral tragedy” to “long-term care tragedy.” Although the average frequency of the term “care burden” increased significantly after 2017, the media largely failed to provide family caregivers with alternative stress-reduction methods or strategies. The media tends to provide more suicide prevention information (eg, hotlines) (>50%), offering relatively limited coverage on long-term care services (<25%). Furthermore, journalists typically used “long-term care” as an adjective or verb to describe care status, rarely specifying duration or treating it as a noun connected to government services or caregiving needs.

The term “long-term care tragedy” first appeared in Taiwan’s news at the end of 2016. The implied case types of such events can be categorized into three types: (1) family caregiver died by suicide after killing care recipient, (2) caregiver perpetrated suicide, and (3) caregiver killed care recipient. Notably, this study found that the suicide rate of family caregivers involved in these reported events was more than 60%, suggesting that suicide prevention efforts by authorities should actively focus on this risk group. In the field of family caregiving, researchers have begun focusing on family caregiver and care-recipient suicides and homicide-suicides [12,13,17]. As O’Dwyer et al [12] already mentioned, risk and protective factors vary across studies, and there is little consideration of differences by caregiving relationship, type of illness or disability, or country. Future research should investigate etiological differences between suicide death by caregivers and homicide of care recipients, and governments should address the impacts of such events and their prevention.

Prior research underscores the critical need for both psychological and practical support for family caregivers [35-37]. However, news headlines indicate that the media’s framework has shifted from “family moral ethics” to the “long-term care” issue. While the reports also mention the stress on family caregivers due to continuing to provide care or the inability to do so, the focus remains on the relationship between family caregivers and care recipients and how the tragic incidents happened, and most do not mention the use of long-term care services by recipients. The media’s reporting framework still appears to attribute the burdens and responsibilities of caregiving to individuals rather than addressing the social or systemic aspects.

Our study found that social service information provided in the news emphasizes suicide prevention over long-term care, often including information irrelevant to the particular case. This may be related to Taiwan’s ongoing implementation of a national suicide prevention strategy since 2005 and national mental health promotion plan since 2013, which have shown remarkable results in preventing suicide. However, this also represents the challenge of people being unable to obtain useful long-term care service information from these news reports. In other words, although the government has introduced policies and laws related to long-term care, it has not yet been able to exert a significant transformational effect on the framing of news media. As Vreese [25] suggested, more studies are needed to explore the framing construction process, including the viewpoints of experts and how they contribute to this process. Even when suicide prevention hotlines are provided, we recommend that such news should avoid detailed descriptions of suicide methods or presenting suicide as a normal reaction to stressful situations. Such news should also include long-term care service application hotlines, counteracting the “Werther effect” with the “Papa Gino effect” by providing readers with clear channels for seeking help.

Taiwan’s National Health Insurance was passed in 1994 and the public is familiar with the procedures for directly accessing most health services, whereas accessing Long-term Care 2.0 service, implemented in 2017, requires calling the service application hotline and having a care manager visit the home to assess the degree of disability. The Multidimensional Assessment Instrument for Long-term Care Disability Assessment has six dimensions: assessment of (1) communication skills or short-term memory, (2) activities of daily living and instrumental activities of daily living, (3) special complex care needs, (4) home environment and social participation, (5) emotional and behavioral patterns, and (6) caregiver burden, work, and support. Results are divided into 8 levels to determine the subsidy provided. These details are seldom reported in news stories, leaving the public less informed about how to access services.

In summary, increasing media understanding of long-term care services, particularly service application hotlines and changes after using services, is an area where the government can focus on helping those in need access the services on offer, as well as increasing awareness of service use. Moreover, this study recommends that relevant government departments work to strengthen media publicity and improve media literacy in long-term care. In addition, it recommends that relevant government agencies establish a database of annual cases detailing family caregiver homicides and suicides that can be used to identify patterns and the underlying factors behind these events, which can be provided to the news media for follow-up reporting or retrospective analysis. This initiative would provide the media with substantive content for their news reports and improve the media’s understanding of the long-term care event discourse.

This study’s findings indicate that caregivers use terms that describe mental disorders or depressive symptoms with significant frequency, which deserves attention. In Taiwan, support services for family caregivers include psychological counseling. Further research is recommended to explore the link between family caregivers’ use of psychological counseling services and their experiences of suicidal or homicidal thoughts.

### Limitations

Taiwan lacks governmental statistics on the annual number of family caregiver homicides and suicides; using news articles to explore the incidence of suicides and homicides in long-term care might result in underestimation. The news media often prioritizes reporting on significant societal events or major issues, sidelining coverage of other news events. Relevant cases might go unreported, and the incidence of suicides and homicides in long-term care might be underestimated. This limitation prevents a thorough exploration of the relationship between the actual case incidence and the number of news articles.

Most news reports emphasize circumstances at the time the incidents occurred. Sometimes, cases initially not covered in the news gain attention only when they enter the judicial process. However, this study found that when both family caregivers and care recipients die simultaneously, and thus the cases do not enter the judicial process, follow-up reporting beyond the initial coverage is limited, and the societal issues underlying these cases warrant continued attention.

### Conclusions

With an aging society, demand for long-term care services continues to rise. Homicides are the only crime that regularly results in the suicide of the perpetrator, an indication that one can be suicidal and homicidal at the same time [16]. It is important for policy makers to recognize that family burden can be perpetrated within an intimate dyad, and traumatic fatalities may result. To date, this research involves gathering and analyzing information about family caregiver homicides and suicides. The evidence suggests that domestic violence has multiple causes, and perpetrating it can encourage others who are at risk to copy maladaptive behaviors.

How the government promotes long-term care policies through the media and whether the public can quickly obtain relevant information are demonstrably influenced by how the media reports them. This study underscores the media’s crucial role in effectively communicating long-term care policies and services. Our findings suggest that the government should strengthen media publicity and improve media literacy in long-term care to encourage media coverage of positive experiences with long-term care services and to provide readers with clear channels for seeking help (eg, hotlines), which will ultimately contribute to the mental health of family caregivers and reduce the incidence of caregiving-related suicides and homicides. It might improve society’s overall health in the face of the challenges posed by an aging population.

In addition, it is crucial to understand and address the caregiving burden and mental health of family caregivers. If the government establishes an annual database that collects information on family caregiver homicide-suicides, the potential risk factors can be identified, and the government can also formulate and revise relevant policies and services to prevent caregiving suicides and homicides in long-term care and achieve public health prevention goals.

## Appendix

Multimedia Appendix 1 Analysis of the difference in the average term frequency of selected vocabulary used in news headlines and articles before December 31, 2016, and after January 1, 2017. The *P* values were derived from a random-effects model.

## Abbreviations

aTF: average term frequency

## Data Availability

All data were gathered by web scraping publicly available news media websites using the keywords “long-term care,” “caregiving tragedies,” “elderly,” and “disabled people” to find relevant news reports. If needed, the datasets used and analyzed during this study are available from the corresponding author upon reasonable request.
