# Supplementary material for: Media Discourse, Influence, and Reflection: Content Analysis and Text-Mining Study of Suicides and Homicides in Long-Term Care
Source: J Med Internet Res. 2025 Apr 28;27:e59037. doi: 10.2196/59037 (PMC12070008; doi:10.2196/59037)
Supplement: Multimedia Appendix 1 [file jmir_v27i1e59037_app1.docx]

A random-effects model was applied to examine whether there were statistically significant differences between the average term frequencies used before 2017 and after.

Table S1. Term frequency (mean $\pm$ SD) of selected vocabulary used in news headlines and testing results for Figure 1.

| Term | Term Frequency | | Regression coefficient | *T* statistic (df = 337) | *P* value |
| --- | --- | --- | --- | --- | --- |
|  | Before 2017 | After 2017 |  |  |  |
| care burden | 0.0006 ± 0.008 | 0.017 ± 0.0461 | 0.0166 | 3.0061 | .0028 |
| death | 0.0590 ± 0.1091 | 0.0298 ± 0.069 | -0.0282 | -2.1652 | .0311 |
| homicide case | 0.0109 ± 0.0599 | 0.0075 ± 0.0333 | -0.0138 | -2.0984 | .0366 |
| suicide | 0.0611 ± 0.1218 | 0.0166 ± 0.0469 | -0.0229 | -1.9690 | .0498 |
| tragedy | 0.0183 ± 0.0619 | 0.0289 ± 0.0579 | 0.0150 | 1.8617 | .0635 |
| shock news | 0 ± 0 | 0.0057 ± 0.0272 | 0.0048 | 1.7133 | .0876 |
| long-term care service | 0 ± 0 | 0.0012 ± 0.0118 | 0.0013 | 1.2974 | .1954 |
| paralysis | 0.0104 ± 0.0518 | 0.0031 ± 0.0217 | -0.0065 | -1.2465 | .2134 |
| care status | 0.0016 ± 0.0144 | 0.005 ± 0.0265 | 0.0031 | 1.1934 | .2335 |
| charcoal-burning | 0.0267 ± 0.0709 | 0.0073 ± 0.0334 | -0.0090 | -1.0364 | .3008 |
| suicide note | 0.0079 ± 0.0376 | 0.0022 ± 0.0169 | -0.0042 | -1.0213 | .3078 |
| hang oneself | 0.0095 ± 0.0396 | 0.005 ± 0.0264 | -0.0054 | -0.9479 | .3439 |
| care | 0.0218 ± 0.0578 | 0.0155 ± 0.0459 | -0.0060 | -0.9450 | .3454 |
| illness | 0.0474 ± 0.0787 | 0.0385 ± 0.0779 | -0.0101 | -0.9202 | .3581 |
| push | 0.0061 ± 0.0366 | 0.0028 ± 0.0191 | -0.0035 | -0.8309 | .4066 |
| kill | 0.0905 ± 0.1234 | 0.0879 ± 0.105 | -0.0131 | -0.7488 | .4545 |
| double death | 0.0176 ± 0.0563 | 0.0116 ± 0.0406 | -0.0058 | -0.7312 | .4651 |
| abuse | 0.0038 ± 0.0277 | 0.0191 ± 0.055 | 0.0039 | 0.6824 | .4955 |
| home | 0.006 ± 0.0339 | 0.0037 ± 0.0221 | -0.0023 | -0.6481 | .5174 |
| a slap on the wrist | 0.0035 ± 0.0265 | 0.0046 ± 0.0279 | 0.0013 | 0.4514 | .6520 |
| cannot bear to | 0.0055 ± 0.0324 | 0.0045 ± 0.0279 | -0.0014 | -0.3928 | .6948 |
| disability | 0.0049 ± 0.0303 | 0.0033 ± 0.0207 | 0.0014 | 0.3773 | .7062 |
| older | 0.0115 ± 0.046 | 0.0106 ± 0.0391 | -0.0021 | -0.3634 | .7166 |
| family moral tragedy | 0.0179 ± 0.0608 | 0.0121 ± 0.0416 | -0.0018 | -0.2707 | .7868 |
| mental disability | 0.0055 ± 0.0454 | 0.0068 ± 0.0328 | -0.0013 | -0.2463 | .8056 |
| stroke | 0.0077 ± 0.0365 | 0.0021 ± 0.0178 | 0.0011 | 0.2368 | .8130 |
| unbearable | 0.0077 ± 0.035 | 0.0067 ± 0.0288 | 0.0007 | 0.1659 | .8684 |
| surrender | 0.0013 ± 0.0159 | 0.005 ± 0.0246 | 0.0004 | 0.1111 | .9116 |
| long-term | 0.0078 ± 0.0342 | 0.0082 ± 0.0324 | 0.0004 | 0.1109 | .9118 |
| cancer | 0.0094 ± 0.0376 | 0.0066 ± 0.0304 | -0.0003 | -0.0585 | .9534 |

Table S2. Term frequency (mean $\pm$ SD) of selected vocabulary used in news articles and testing results for Figure 2.

| Term | Term Frequency | | Regression coefficient | *T* statistic (df = 337) | *P* value |
| --- | --- | --- | --- | --- | --- |
|  | Before 2017 | After 2017 |  |  |  |
| Euthanasia | 0.0005 ± 0.0042 | 0.0004 ± 0.0042 | 0.0022 | 3.4061 | .0007 |
| filial piety | 0.0032 ± 0.0095 | 0.0002 ± 0.0014 | -0.0025 | -2.8489 | .0047 |
| care burden | 0.0005 ± 0.0022 | 0.0016 ± 0.0046 | 0.0012 | 2.5974 | .0098 |
| placement | 0.0005 ± 0.0025 | 0 ± 0.0004 | -0.0005 | -2.5417 | .0115 |
| suicide | 0.0165 ± 0.0187 | 0.0092 ± 0.015 | -0.0063 | -2.4566 | .0145 |
| seek medical care | 0.0004 ± 0.0017 | 0.0013 ± 0.0039 | 0.0009 | 2.2386 | .0258 |
| treatment | 0.0007 ± 0.0026 | 0.0027 ± 0.0064 | 0.0014 | 2.1331 | .0336 |
| discharge | 0.0001 ± 0.0007 | 0.0005 ± 0.0021 | 0.0005 | 2.0529 | .0409 |
| domestic violence | 0.0001 ± 0.0011 | 0.0016 ± 0.0051 | 0.0015 | 1.9446 | .0527 |
| home service | 0.0007 ± 0.0023 | 0.0003 ± 0.0016 | -0.0005 | -1.9206 | .0556 |
| Department of Health Welfare | 0 ± 0 | 0.0004 ± 0.0023 | 0.0004 | 1.8772 | .0614 |
| drag | 0.0007 ± 0.0033 | 0.0001 ± 0.0011 | -0.0008 | -1.8647 | .0631 |
| residential institution | 0.0019 ± 0.0054 | 0.0004 ± 0.0024 | -0.0010 | -1.5831 | .1143 |
| poverty | 0.0003 ± 0.0014 | 0.0003 ± 0.0017 | 0.0004 | 1.5420 | .1240 |
| cerebral palsy | 0.0002 ± 0.0018 | 0.0013 ± 0.006 | 0.0012 | 1.5132 | .1312 |
| medical | 0.0001 ± 0.001 | 0.0003 ± 0.0016 | 0.0002 | 1.4789 | .1401 |
| depression | 0.0024 ± 0.007 | 0.0011 ± 0.0045 | -0.0015 | -1.4698 | .1425 |
| outbreak | 0.0003 ± 0.0018 | 0.0002 ± 0.0012 | -0.0003 | -1.4537 | .1470 |
| patient | 0.0015 ± 0.0051 | 0.0009 ± 0.0039 | -0.0007 | -1.4328 | .1528 |
| care | 0.0174 ± 0.0188 | 0.0136 ± 0.0144 | -0.0037 | -1.4078 | .1601 |
| long-term care service | 0.0007 ± 0.0028 | 0.0016 ± 0.0057 | 0.0009 | 1.3812 | .1681 |
| schizophrenia | 0 ± 0 | 0.001 ± 0.0038 | 0.0005 | 1.3764 | .1696 |
| foreign care worker | 0.0018 ± 0.0065 | 0.0007 ± 0.0029 | -0.0012 | -1.3628 | .1739 |
| care worker | 0.0019 ± 0.0058 | 0.0013 ± 0.0062 | -0.0010 | -1.3293 | .1847 |
| diabetes | 0.0003 ± 0.0016 | 0.0009 ± 0.0033 | 0.0006 | 1.2446 | .2141 |
| chronic disease | 0.0001 ± 0.001 | 0.0006 ± 0.0024 | 0.0003 | 1.2260 | .2211 |
| rehabilitation | 0.0003 ± 0.0018 | 0.0001 ± 0.0008 | -0.0002 | -1.1778 | .2397 |
| low-income family | 0.0001 ± 0.0013 | 0.0005 ± 0.0027 | 0.0004 | 1.1383 | .2558 |
| abandon | 0.0019 ± 0.0091 | 0 ± 0 | -0.0009 | -1.1312 | .2588 |
| failure | 0.0001 ± 0.0008 | 0.0003 ± 0.0016 | 0.0002 | 1.1299 | .2593 |
| burden | 0.0004 ± 0.0022 | 0.0003 ± 0.0016 | -0.0002 | -1.0984 | .2728 |
| epilepsy | 0.0007 ± 0.0038 | 0.0001 ± 0.0013 | -0.0004 | -1.0509 | .2941 |
| care status | 0.0026 ± 0.0058 | 0.0031 ± 0.0068 | 0.0010 | 1.0396 | .2993 |
| physical disability | 0.0007 ± 0.0028 | 0 ± 0.0003 | -0.0003 | -1.0060 | .3151 |
| intellectual disability | 0.0005 ± 0.0019 | 0.0006 ± 0.0035 | 0.0005 | 0.9867 | .3245 |
| family moral tragedy | 0.0025 ± 0.0061 | 0.0019 ± 0.0047 | -0.0007 | -0.9779 | .3288 |
| patients' condition | 0.0009 ± 0.0038 | 0.0007 ± 0.0029 | -0.0004 | -0.9746 | .3305 |
| paralysis | 0.0011 ± 0.0046 | 0.0005 ± 0.003 | -0.0005 | -0.9741 | .3307 |
| illness | 0.016 ± 0.0163 | 0.0137 ± 0.0148 | -0.0023 | -0.9606 | .3374 |
| hospital | 0.0108 ± 0.0194 | 0.0065 ± 0.0103 | -0.0021 | -0.9581 | .3387 |
| taking things too hard | 0.001 ± 0.0035 | 0.0004 ± 0.0018 | -0.0004 | -0.8548 | .3933 |
| out of control | 0.0011 ± 0.0044 | 0.0018 ± 0.0051 | 0.0006 | 0.8233 | .4109 |
| social worker | 0.0004 ± 0.0016 | 0.0005 ± 0.0018 | 0.0002 | 0.7886 | .4309 |
| seek help | 0.0007 ± 0.0024 | 0.0004 ± 0.002 | -0.0002 | -0.7878 | .4314 |
| extubation | 0.0006 ± 0.0036 | 0.0001 ± 0.0017 | -0.0003 | -0.7793 | .4364 |
| part-time job | 0.0003 ± 0.0018 | 0.0004 ± 0.0019 | 0.0003 | 0.7766 | .4380 |
| auditory hallucination | 0 ± 0.0002 | 0.0003 ± 0.0017 | 0.0001 | 0.7645 | .4451 |
| unemployment | 0.0012 ± 0.0036 | 0.0011 ± 0.0041 | 0.0005 | 0.7541 | .4513 |
| take home | 0.0005 ± 0.0028 | 0.0002 ± 0.0013 | -0.0002 | -0.7466 | .4558 |
| unfilial | 0.0002 ± 0.0011 | 0.0005 ± 0.0029 | 0.0003 | 0.7381 | .4610 |
| abuse | 0.0013 ± 0.0044 | 0.0027 ± 0.0063 | 0.0005 | 0.7151 | .4750 |
| debt | 0.0003 ± 0.0017 | 0.0003 ± 0.0022 | -0.0002 | -0.6885 | .4916 |
| older adult | 0.0069 ± 0.0126 | 0.0047 ± 0.0097 | -0.0010 | -0.6075 | .5439 |
| application | 0.0008 ± 0.0028 | 0.001 ± 0.0038 | 0.0002 | 0.5837 | .5598 |
| double death | 0.002 ± 0.0055 | 0.0015 ± 0.0051 | -0.0004 | -0.5620 | .5745 |
| depend on each other | 0.0001 ± 0.0011 | 0.0002 ± 0.0015 | 0.0001 | 0.5568 | .5780 |
| mental | 0.0024 ± 0.0072 | 0.004 ± 0.0083 | 0.0006 | 0.5535 | .5803 |
| hypertension | 0.0003 ± 0.0018 | 0.0006 ± 0.0029 | 0.0002 | 0.5504 | .5824 |
| the last phase | 0.0005 ± 0.0027 | 0.0005 ± 0.0026 | 0.0002 | 0.5216 | .6023 |
| critically ill | 0.0006 ± 0.004 | 0 ± 0 | -0.0001 | -0.4982 | .6187 |
| disability | 0.0022 ± 0.0063 | 0.0025 ± 0.007 | 0.0005 | 0.4976 | .6191 |
| Department of Social Welfare | 0.0013 ± 0.0048 | 0.0011 ± 0.0046 | -0.0003 | -0.4970 | .6195 |
| handicapped | 0.001 ± 0.0046 | 0.0011 ± 0.0043 | -0.0003 | -0.4818 | .6303 |
| clinic | 0.0001 ± 0.0009 | 0.0003 ± 0.0018 | 8.413$\times{10}^{-5}$ | 0.4518 | .6517 |
| social welfare | 0.0002 ± 0.0014 | 0.0003 ± 0.0016 | 9.789$\times{10}^{-5}$ | 0.4370 | .6624 |
| worse | 0.0003 ± 0.0015 | 0.0004 ± 0.0022 | 0.0001 | 0.4064 | .6847 |
| unable to move freely | 0.0017 ± 0.0044 | 0.0016 ± 0.0044 | -0.0003 | -0.4014 | .6884 |
| heart disease | 0.0002 ± 0.0014 | 0.0003 ± 0.0016 | -0.0001 | -0.3613 | .7181 |
| cancer | 0.0024 ± 0.0067 | 0.0017 ± 0.0053 | 0.0003 | 0.2962 | .7672 |
| exhausted | 0.0004 ± 0.0026 | 0.0006 ± 0.0028 | 0.0001 | 0.2854 | .7755 |
| have no way out | 0.0008 ± 0.0027 | 0.0003 ± 0.0018 | -0.0001 | -0.2783 | .7810 |
| physical and mental | 0.0016 ± 0.0042 | 0.0018 ± 0.0044 | 0.0001 | 0.2407 | .8099 |
| bed ridden | 0.0024 ± 0.0055 | 0.0029 ± 0.007 | 0.0003 | 0.2282 | .8196 |
| allowance | 0.001 ± 0.0037 | 0.0011 ± 0.0038 | -0.0001 | -0.1744 | .8616 |
| economy | 0.0018 ± 0.0039 | 0.0013 ± 0.004 | -0.0001 | -0.1619 | .8715 |
| service | 0.0016 ± 0.0053 | 0.0017 ± 0.0072 | -0.0001 | -0.1405 | .8883 |
| bipolar disorder | 0.0002 ± 0.0014 | 0.0004 ± 0.0019 | 2.927$\times{10}^{-5}$ | 0.1247 | .9008 |
| hospice care | 0.0001 ± 0.0009 | 0 ± 0.0006 | -1.196$\times{10}^{-5}$ | -0.1111 | .9116 |
| mental exhaustion | 0.0002 ± 0.0015 | 0.0002 ± 0.0014 | -1.188$\times{10}^{-5}$ | -0.0794 | .9367 |
| hypnotics | 0.0004 ± 0.003 | 0.0003 ± 0.0021 | -1.876$\times{10}^{-5}$ | -0.0655 | .9478 |
| fatigue | 0.0013 ± 0.0035 | 0.0014 ± 0.0041 | 1.749$\times{10}^{-5}$ | 0.0347 | .9723 |
| respite service | 0.0007 ± 0.0029 | 0.0009 ± 0.0041 | 1.230$\times{10}^{-5}$ | 0.0286 | .9772 |
| mental disability | 0.0004 ± 0.0027 | 0.0006 ± 0.0031 | 3.943$\times{10}^{-5}$ | 0.0106 | .9915 |
| dementia | 0.0034 ± 0.0085 | 0.0023 ± 0.0067 | -8.277$\times{10}^{-6}$ | -0.0070 | .9944 |
